# Repeatability and reproducibility of quantitative cervical strain elastography (E-Cervix) in pregnancy

**DOI:** 10.1038/s41598-021-02498-3

**Published:** 2021-12-08

**Authors:** Jakub Mlodawski, Marta Mlodawska, Justyna Plusajska, Karolina Detka, Agata Michalska, Grzegorz Swiercz, Marek Sikorski

**Affiliations:** 1 Collegium Medicum, Jan Kochanowski University in Kielce, Kielce, Poland; 2 Clinic of Obstetrics and Gynaecology, Provincial Combined Hospital in Kielce, Kielce, Poland

**Keywords:** Preclinical research, Reproductive signs and symptoms

## Abstract

Strain elastography of the uterine cervix may be useful in the diagnosis and prediction of obstetric complications. The inability to obtain quantitative results, with only the possibility of visual semiquantitative evaluation of the obtained elastograms, has been the limitation of the method thus far. E-Cervix is a software program that uses intrinsic compression to excite tissue and allows the evaluation of quantitative parameters on the basis of pixel distribution in an elastogram. The aim of this study was to assess the repeatability and reproducibility of quantitative cervical strain elastography (E-Cervix) of the uterine cervix and to assess the correlation of the obtained parameters with selected clinical features of patients in the third trimester of pregnancy. In total, 222 patients participated in the study. We assessed 5 ultrasound parameters: elasticity index (ECI), hardness ratio (HR), internal os strain (IOS), external os strain (EOS) and IOS/EOS ratio. Each study was performed according to a predetermined standardized protocol. For all assessed elastographic parameters, we obtained good intra- and interobserver reproducibility. The interclass correlation coefficient (ICC) ranged from 0.77 to 0.838 for intraobserver variability and from 0.771 to 0.826 for interobserver variability. We demonstrated a significant correlation of some obtained elastographic parameters with the basic clinical features of patients, such as age, the number of previous caesarean sections, pregnancy weight and BMI. In each case, the correlation was very low. Quantitative elastographic assessment with the use of E-Cervix is characterized by good repeatability. Some clinical features may affect the value of the parameters obtained. The clinical relevance of this interference requires further investigation.

## Introduction

Ultrasound elastography (USE) of human organs has already found its use in many medical specializations. USE is used, among other applications, in the evaluation of the fibrosis process affecting the liver and transplanted kidneys. In the case of focal lesions in the breast, thyroid, liver and prostate, USE can be used as a supplement to other imaging techniques, allowing for better classification of radiological changes and a reduction in the percentage of organ biopsies^1^. In obstetrics, it seems natural to employ USE to evaluate the consistency of the cervix. Cervical hardness is a standard element assessed in the bimanual examination of the pelvis during pregnancy, and it is also an element of the Bishop scale assessment^2^. Consistency examination can be used in the prediction of preterm labour^3,4^, in the prediction of a spontaneous onset of labour and in evaluations of the effectiveness of the induction of labour (IOL)^5,6^. The two main techniques of USE are strain elastography (SE) and shear wave elastography (SWE), differing in terms of the stress type applied to tissue.

To date, SE has been limited by its presentation of results only in the form of an elastogram, i.e., a "colour map" of the organ, in which the intensity of the colour corresponds to the stiffness of the organ (qualitative assessment). The elastogram can then be visually assessed with the possible use of semiquantitative scales^7^. The E-Cervix (Samsung Medison), a semiautomatic system for the elastographic evaluation of the cervix, meets the expectations of the quantitative approach to the elastogram. In this method, the cervix is examined with the use of SE—in which the necessary vibrations of the tissue are excited by intrinsic factors—mainly due to the pulse wave of the pelvic vessels carried by the surrounding tissues^8^. As part of the test results, in addition to the colour elastogram, we also obtained measurable quantitative parameters calculated on the basis of the pixel colour distribution within the elastogram. The parameters obtained, together with their definitions, are listed in Table 1.

**Table 1:** Description of E-Cervix parameters^8,9^.

E-cervix parameter	Description
ECI (Elasticity index)	A measure of tissue heterogeneity. It informs about the average difference in colour intensity between neighbouring pixels of the elastogram and adopts values from 0 to 81 (0-low heterogeneity, 81-high heterogeneity)
HR (Hardness ratio)	The number of red pixels (defined as the top 30% of the colour intensity scale) among all of the pixels in the ROI. This value is displayed as a percentage (0%-soft, 100%-hard)
IOS (Internal Os strain)	Mean strain level of the internal cervical os ROI [region of interest] (0-hard, 1-soft)
EOS (External Os strain)	Mean strain level of the external cervical os ROI (0-hard, 1-soft)
Ratio (IOS/EOS)	Ratio of the internal to the external cervical os mean strain
CL (cervical length)	Length of the cervical canal

### Objective

The study was intended to assess the repeatability and reproducibility of the parameters obtained in the E-Cervix examination.

The secondary aim was to correlate the obtained parameters with the basic demographic data of the female patients.

## Methods

Female patients in their third trimester of pregnancy hospitalized in the Pregnancy Pathology Department of the Gynaecology and Obstetrics Clinic of the Provincial Combined Hospital in Kielce in 2020 (third degree of referentiality) participated in the study. We received approval to examine the patients for research purposes from the bioethics commission at Jan Kochanowski University in Kielce (approval number—55/2019). All methods were performed in accordance with the relevant local regulations and guidelines of the ethical commission. All participants gave informed consent for ultrasonographic diagnosis and participation in the study. In this study, we used a Samsung WS80A ultrasound device with a 6 MHz vaginal ultrasound transducer and E-Cervix software.

**Figure 1:**
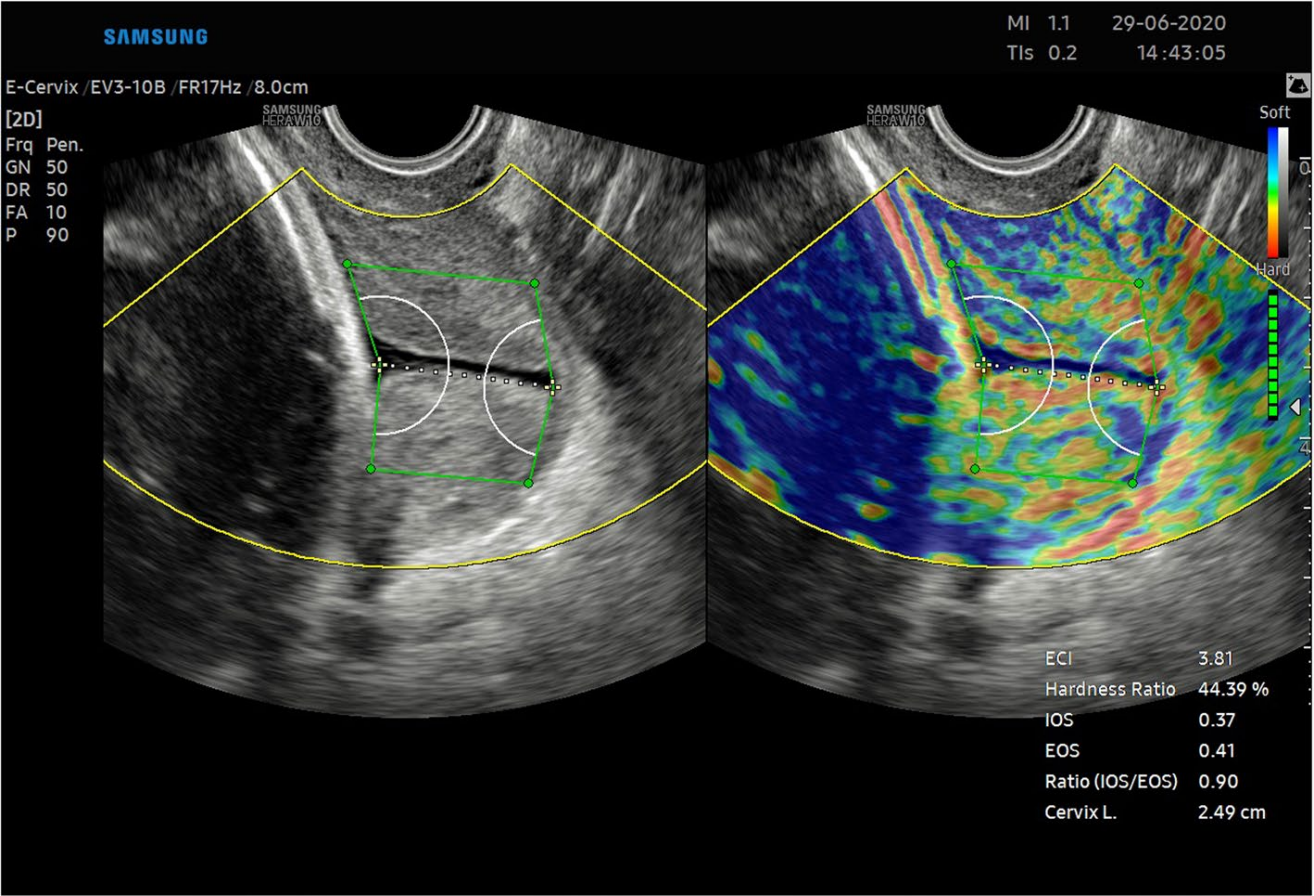
Sample image of the cervix obtained in the study. (**a**) The dashed white line represents the endocervical canal; (**b**) the internal and external cervical os are represented by the white circular sectors; and (**c**) the green 6-point sector represents the ROI. In the lower right corner, you can see the values ​​of the E-Cervix parameters.

The demographic characteristics of the sample group are presented in Table 2. Since the study was intended to assess a diagnostic method, the patients were not selected according to the reason for hospitalization. Patients with cervical Naboth cysts, multiple pregnancies, cervical procedures (conization, loop electrosurgical excision procedure), ruptured membranes, cervical cerclage or obstetrical pessary placement, and no clear visualization of internal cervical os were excluded from the study. Two ultrasound specialists were involved in the study. For the assessment of interobserver variability, two specialists performed E-Cervix examination according to a standard protocol immediately after each other, each blinded to the other operator's result. Intraobserver variability was assessed by having the same ultrasound specialist perform the examination two times. The ultrasound transducer was removed from the vagina between the examinations. The protocol of the examination is presented in Table 5. To standardize and compare results from different centres, we used an examination protocol based on the method presented by Seol et al*.*^10^ An example of ultrasound images with elastograms and ROIs is shown in Fig. 1. We compared the quantitative E-Cervix parameters obtained (description in Table 1).

**Table 2:** Baseline characteristics of patients included in the study [*IQR* interquartile range, *SD* standard deviation, *kg* kilograms, *m* metres, *CS* caesarean section].

Parameter	Value
Age (years) [mean, SD]	29.44 (5)
Multipara [n, %]	80 (36)
Gestational age (weeks) [median, IQR]	38 (9)
Weight (kg) [median, IQR]	74 (13)
BMI (kg/m2) [median, IQR]	44.44 (5)
height (m) [median, IQR]	1.66 (0.06)

**Figure 2:**
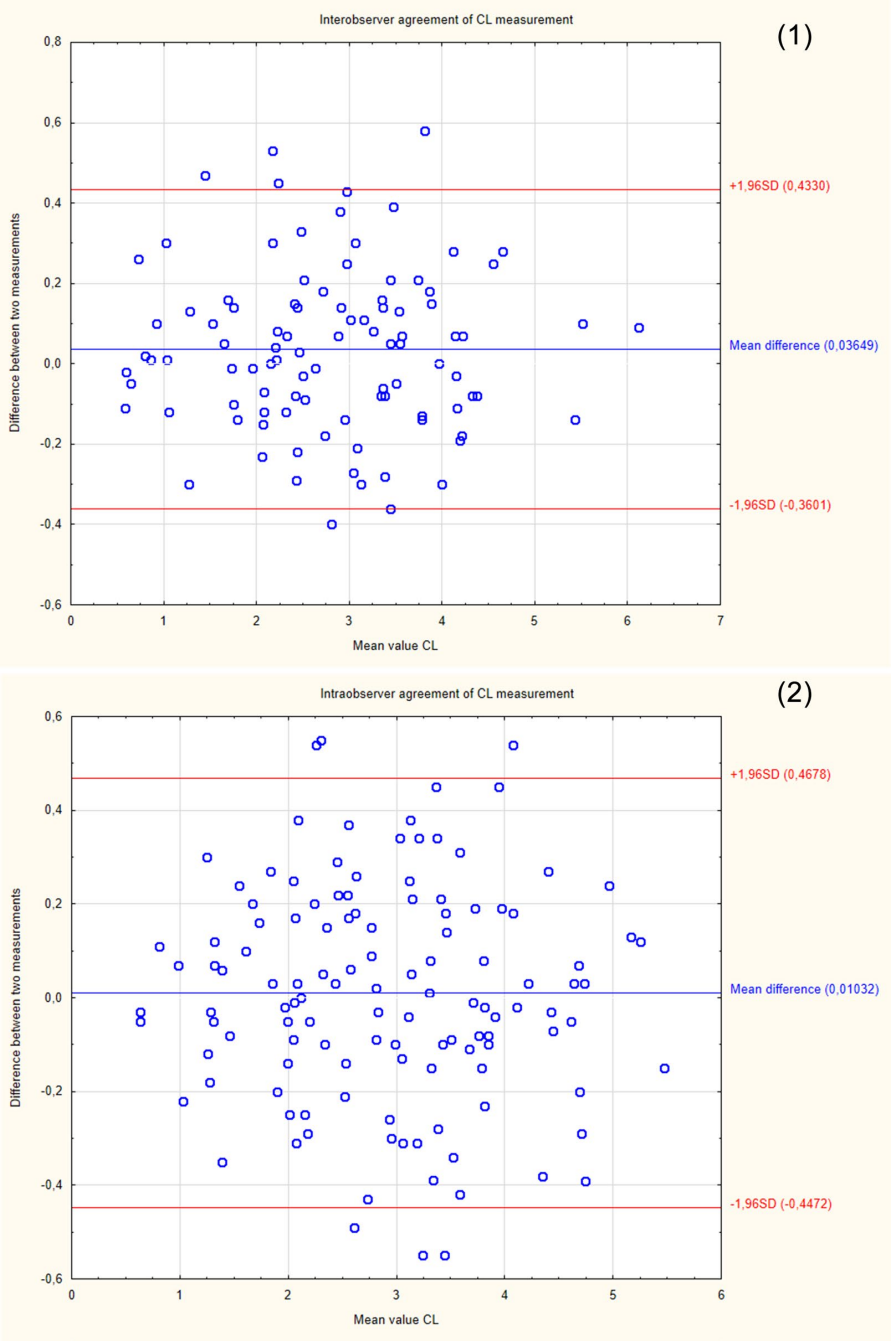

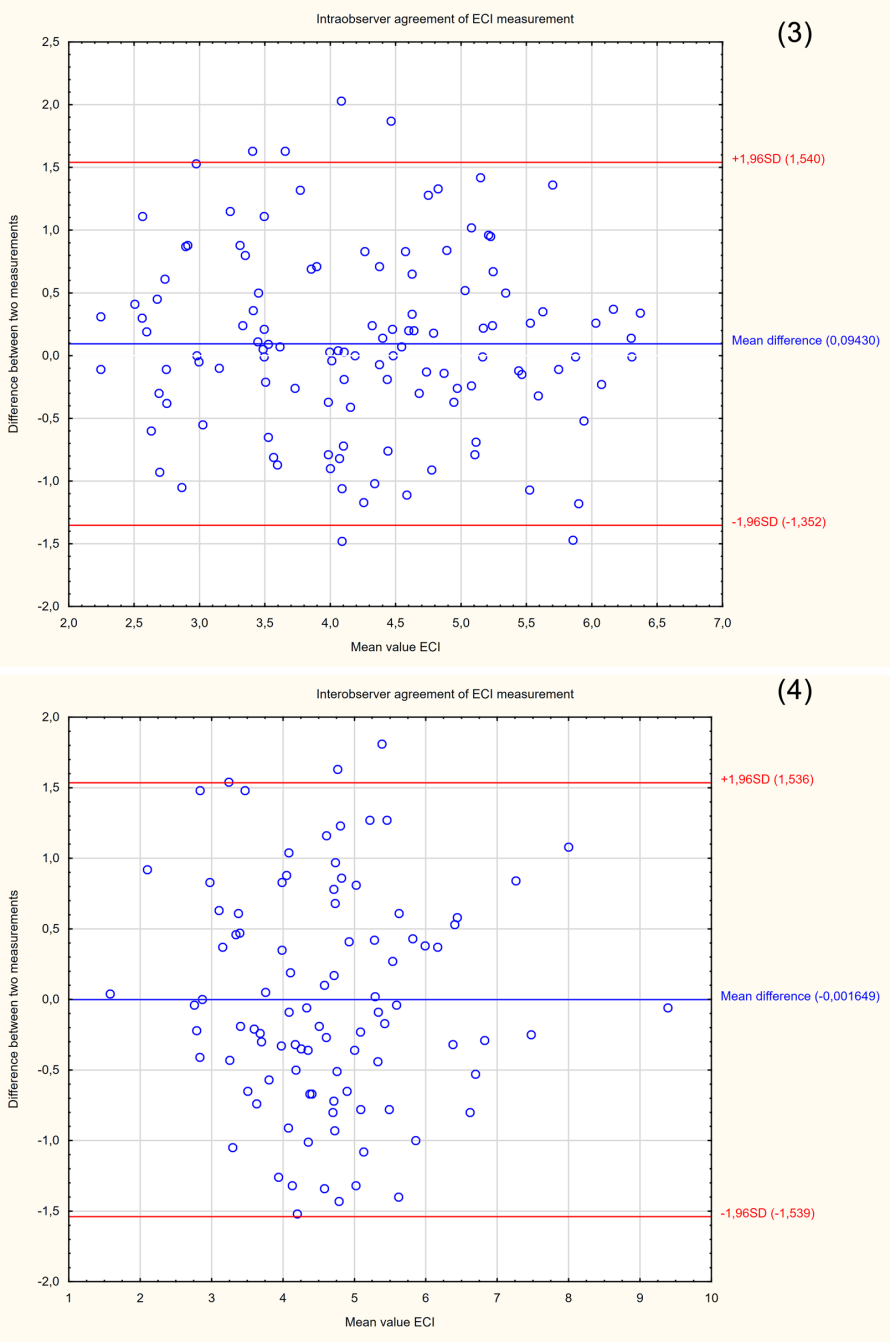

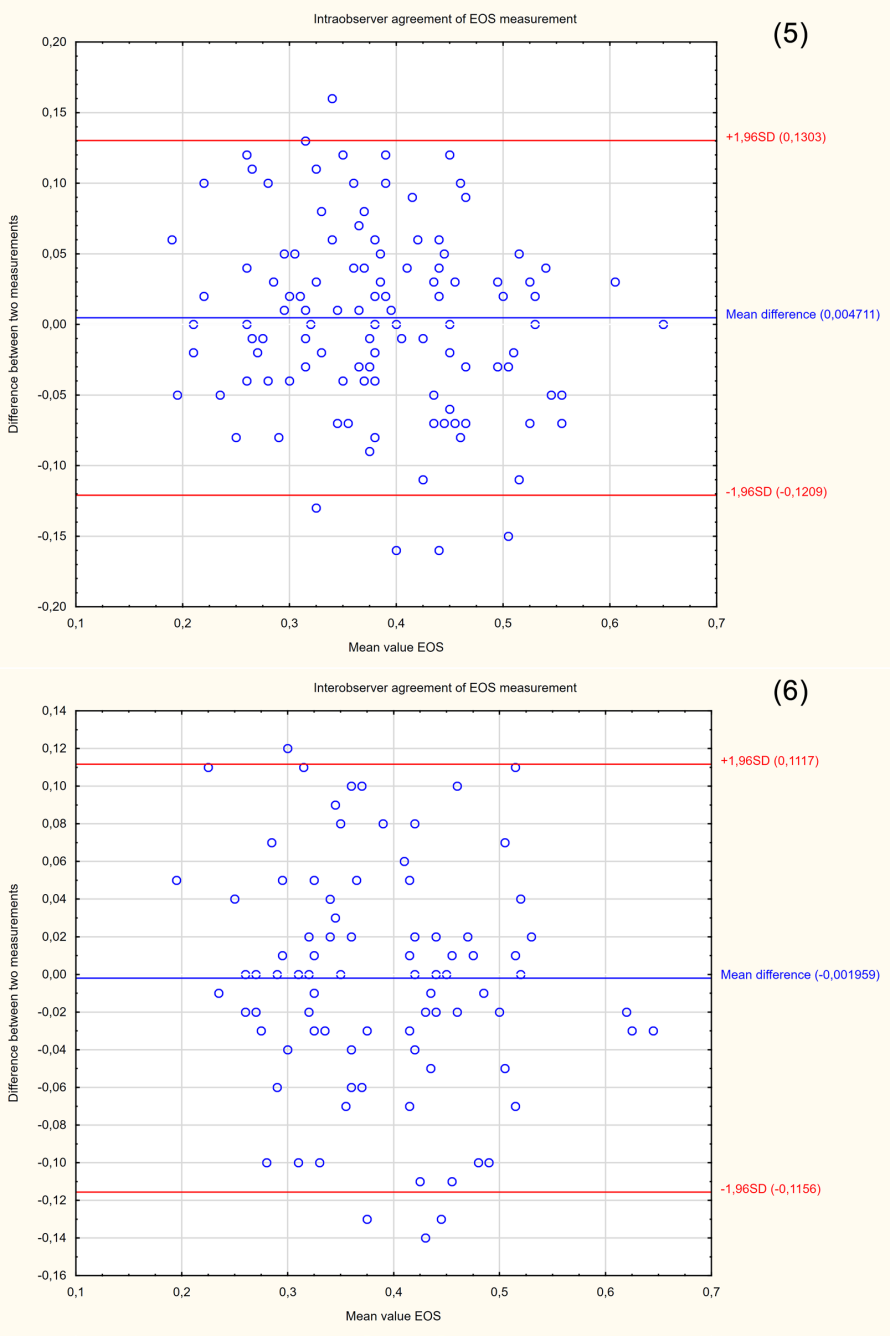

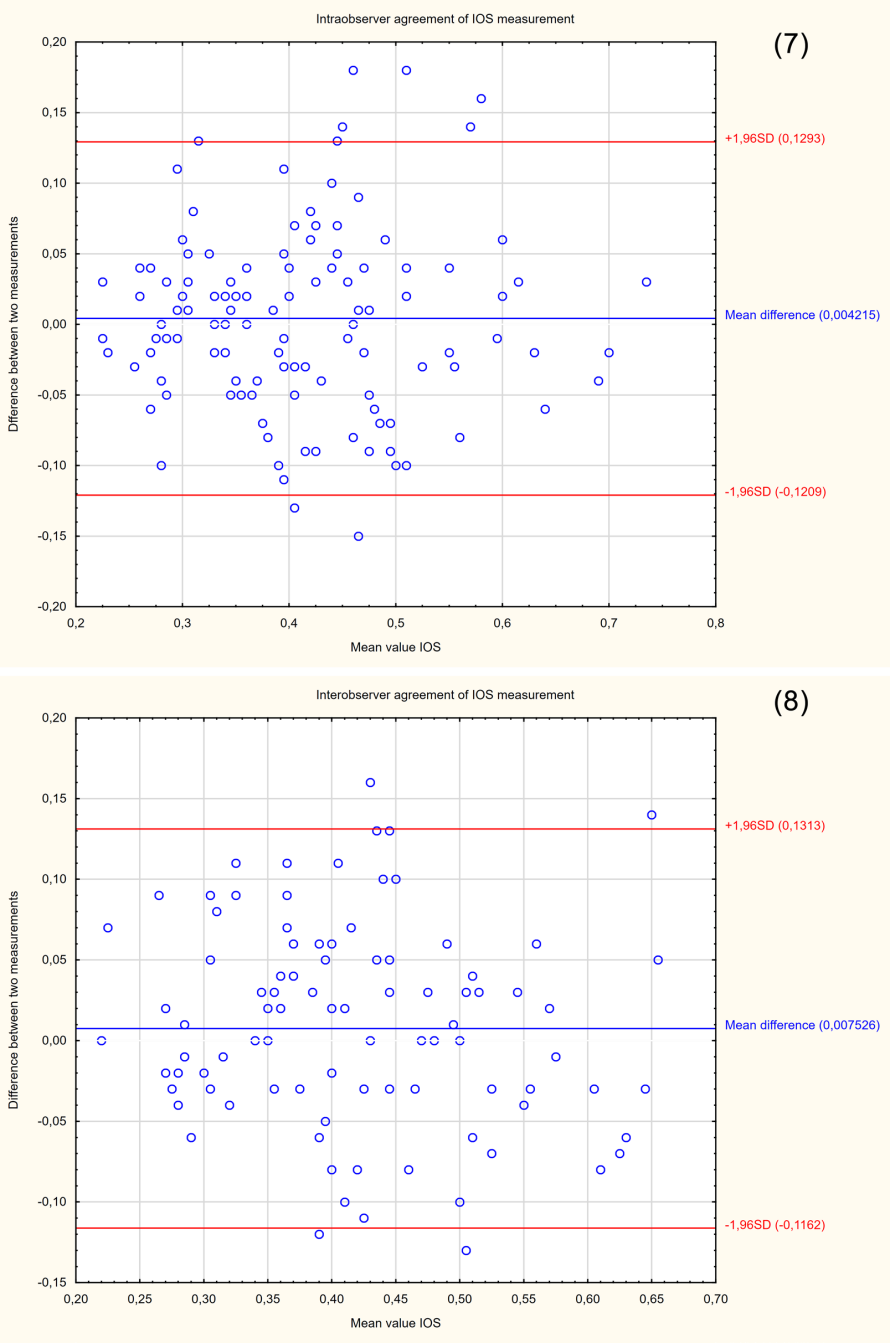

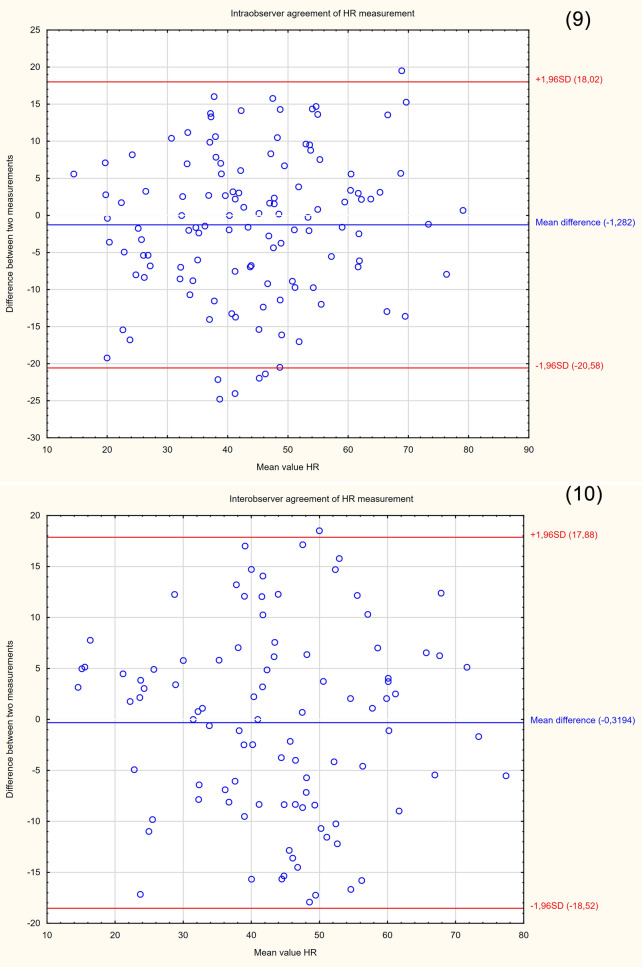
Bland–Altman plots showing the degree of agreement between two examinations with respect to measuring individual E-Cervix parameters. Intrarater agreement and interrater agreement of cervical length (1,2) ECI (3,4), EOS (5,6), IOS (7,8) and HR (9,10) measurements.

### Statistical analysis

For the description of the group, we used the median as a measure of the central tendency for a distribution different from normal (*p* < 0.05 in the Shapiro–Wilk test) and the arithmetic mean for a distribution close to normal. As a measure of scatter, the interquartile range (IQR) and the standard deviation (SD) were employed. To evaluate the intra- and interobserver reproducibility of elastographic cervical parameters, the interclass correlation coefficient (ICC) was calculated. Based on the available literature, it was found that a certain level of interrater and intrarater agreement already exists (in none of the ICC studies found was it less than 0.5)^9,12^. We estimated a minimal sample size based on the table presented by Bujang et al*.*^13^ (with a prespecified null hypothesis set at 0.5 and a prespecified minimal alternative hypothesis set at 0.7, alpha = 0.05 and power = 0.9). Based on these calculations, the minimal sample size to properly assess the given ICC was 87 subjects. The ICC values were interpreted as follows: > 0.9—excellent reproducibility, 0.75–0.9—good reproducibility, 0.5–0.75—moderate reproducibility, and < 0.5—poor reproducibility. Baseline patient parameters were correlated with those assessed in the E-Cervix. In the correlation analysis, the arithmetic mean of two obtained measurements was used. Spearman's rank correlation coefficient (Spearman's rho) was used to assess the correlation. Statistical analysis was performed with SPSS 27.0.1.0 (IBM Company) and Statistica 13.1 (Tibco Software). A *p* value < 0.05 was considered to be statistically significant.

## Results

A total of 222 Caucasian patients were enrolled in the study: 125 patients in the group assessing intraobserver variability (79 for rater A and 46 for rater B) and 97 patients in the group assessing interobserver variability (each patient assessed by raters A and B). The demographic characteristics of the patients and the correlation with the individual elastographic parameters are presented in Table 3. We found a very weak positive correlation between age, the number of previous caesarean sections (CSs) and HR (0.14 and 0.16, respectively); a very weak negative correlation between BMI and ECI (− 0.17); and a very weak positive correlation between BMI and IOS (0.15). Moreover, we observed a weak positive correlation between IOS/EOS and patient weight (0.21) and a very weak negative correlation between EOS and patient age (− 0.15). A moderate negative correlation between cervical length and gestational age (− 0.39) was also found. The remaining elastographic parameters did not significantly correlate with the baseline characteristics of the patients.

**Table 3:** Correlation of E-cervix parameters with baseline patient characteristics [explanation of E-Cervix abbreviations in Table 1].

	ECI [mean]		HR [mean]		IOS [mean]		EOS [mean]		IOS/EOS [mean]		CL [mean]	
	rho correlation	*p*	rho correlation	*p*	rho correlation	*p*	rho correlation	*p*	rho correlation	*p*	rho correlation	*p*
Age [years]	− 0.12	0.09	0.14	0.03	− 0.11	0.11	− 0.15	0.03	0.01	0.93	0.09	0.17
Gravidity [n]	− 0.12	0.08	0.13	0.06	− 0.11	0.12	− 0.13	0.06	0.00	0.97	− 0.03	0.70
Parity [n]	− 0.12	0.09	0.12	0.08	− 0.04	0.53	− 0.13	0.06	0.07	0.28	0.07	0.31
Number of previous CS [n]	− 0.05	0.48	0.16	0.02	− 0.09	0.19	− 0.10	0.14	0.00	0.96	0.07	0.33
Gestational age [weeks]	− 0.06	0.39	− 0.08	0.25	0.12	0.08	0.04	0.54	0.11	0.09	− 0.39	0.00
Weight [kg]	− 0.14	0.06	− 0.02	0.84	0.14	0.07	− 0.05	0.48	0.22	0.00	− 0.03	0.67
Height [cm]	0.09	0.21	− 0.03	0.66	− 0.01	0.93	− 0.07	0.36	0.08	0.25	− 0.05	0.46
BMI [kg/m2]	− 0.17	0.02	− 0.07	0.38	0.15	0.05	0.03	0.73	0.13	0.10	0.02	0.75

The repeatability and reproducibility of quantitative E-Cervix parameters are shown in Table 4. All ICCs received in the intra- and interobserver variability categories were in a good range of reproducibility. The cervical length measurement showed excellent reproducibility. The Bland–Altman plots are shown in Fig. 2. The lowest consistency in terms of repeatability was obtained in the IOS measurement, where 7 (5.6%) of the measurement pairs were outside the level of agreement (LoA—mean of difference ± 1.96 of standard deviation). The lowest agreement in reproducibility was obtained for the EOS measurement, where 4 (4.1%) pairs of measurements were outside the LoA (Table 4., Fig. 1).

**Table 4:** Inter- and intrarater interclass correlation coefficients (ICCs) of E-Cervix parameters [explanation of abbreviations in Table 1].

Parameter	Intraobserver variability			Interobserver variability		
	ICC	95% CI	*p*	ICC	95% CI	*p*
ECI	0.77	0.69–0.83	< 0.001	0.83	0.75–0.88	< 0.001
Hardness ratio	0.77	0.69–0.84	< 0.001	0.79	0.71–0.88	< 0.001
IOS	0.84	0.78–0.88	< 0.001	0.83	0.75–0.88	< 0.001
EOS	0.79	0.71–0.84	< 0.001	0.82	0.74–0.88	< 0.001
IOS/EOS	0.78	0.69–0.84	< 0.001	0.77	0.68–0.84	< 0.001
Cervix lenght	0.98	0.98–0.99	< 0.001	0.98	0.98–0.99	< 0.001

**Table 5:** Protocol for E-cervix measurement. (based on the protocol presented by Seol et al.^10^, with modification by the present authors).

Protocol
1. The mother’s bladder should be empty prior to examination
2. Image orientation—The apex of the image should be displayed at the top of the monitor, and the foetal part is displayed on the left side of the image sector
3. Activation of the E-cervix program and obtaining an optimal cervical image The image plane used for cervical elastography is the same as the one used for measuring cervical length (according to Fetal Medicine Foundation guidelines^11^) without applying pressure with the probe onto the anterior cervix
4. Acquisition of cervical strain: After optimal cervical image acquisition, the probe should be held still until all motion bars (reliability indicator) turn green (use the auto-freeze setting for motion bars) The patient should breathe normally during the acquisition The image should be discarded when active foetal movements occur during the acquisition, especially foetal limb movement in the breech presentation, as this may affect cervical strain
5. ROI (region of interest) calliper placement for strain measurement 1) Callipers are placed on the greyscale image displayed on the left side of the screen, as the elastographic image displayed on the right may be blurred at the margin 2) By selecting either a 2- or 4-point ROI, a line should be drawn along the endocervical canal between the internal and external os of the cervix. If the endocervical line is straight, a 2-point ROI tool should be used. With a curved cervix, a 4-point ROI should be used to trace the endocervical lining to the best extent possible 3) After the cervical canal is defined, green points will automatically appear Place the points on the 4 corner edges of the cervix so that the ROI box includes the entire cervix area. The entire cervix should be included without adjacent structures such as the bladder or vaginal wall

## Discussion

The results of our research show that the semiautomatic system for cervical elastography measurement is characterized by high repeatability. This is also confirmed by the data available in the literature. In none of the studies available in the literature was the ICC of any elastographic parameter lower than 0.633^10,12,14^. EOS was the parameter with the lowest repeatability in each of the available studies. This parameter has the greatest sensitivity to the measurement conditions due to the proximity of the external outlet of the external uterine os to the ultrasound transducer head. The influence of measurement conditions on the EOS value is confirmed by clinical trials with gradual compression of the ultrasound transducer head on the uterine cervix. The EOS value significantly decreases with increasing pressure of the transducer head^12^.

In the study conducted by the Korean Research Group of Cervical Elastography^10^, moderate to excellent reproducibility was achieved depending on the elastographic parameter tested. The intraobserver ICC ranged from 0.633 to 0.723, and the interobserver ICC ranged from 0.814 to 0.977. In our study, we used the same measurement protocol (measurement conditions, ROI determination), whereas the differences concerned the postprocessing method during the database creation. In our study, we wanted the conditions to be closer to real-world settings. We wanted to evaluate the reproducibility of the elastographic parameters directly obtained in research by operators using the same measurement protocol, performing the entire research procedure again.

Seul et al*.*^10^ used external quality control, rejecting measurements with inadequate images (9.8%), while in some cases, remeasurement was used by an expert using precaptured elastograms (10%). Similarly, for the interobserver variability assessment, the second operator used precaptured images to calculate elastographic parameters without retesting. Our study seems to be more adapted to clinical conditions. In each case, the second operator, blinded to the results of the first measurement, performed the examination from the beginning after removing and reinserting the ultrasound transducer into the vagina. In theory, the lack of external control of elastograms should result in less agreement among observers, but nevertheless, the repeatability obtained in our study is equally high. In our opinion, this is evidence of high repeatability when testing is performed according to strictly defined procedures included in a standardized protocol.

One of the variables having a considerable impact on the data obtained in elastography is the degree of pressure of the transducer head on the examined organ and vibrations of the operator's hand transmitted to the head, which results directly from the measurement method (free-hand ultrasound system). Direct pressure of the ultrasound transducer head against the uterine cervix affects both its length and the parameters of the elastogram^12^. The analysis of the pairs of measurements outside the level of agreement from our study shows differences in the distance between the transducer head and the uterine cervix canal. Excessive pressure on the transducer head, in addition to increasing the cervical length, also affects the increase in the HR value and the decrease in the EOS value and thus the increase in the IOS/EOS ratio^12^.

Therefore, it seems that the key to achieving high reproducibility is the unification of the rules for transducer head placement with the recommendations for cervical measurements in the second trimester of pregnancy^15^ and the standard protocol described in the literature (also used in our study)^10^. This procedure will allow for the comparison of the results of multicentre studies and research in the application of E-Cervix in clinical practice. The influence of the transducer head movements on the obtained values, in the case of the E-Cervix software, is limited by the reliability indicator. This indicator is a stack of colour blocks displaying acquisition reliability. In the autofreeze mode, the image freezes only when the ultrasound device obtains a sufficient number of steady frames to provide satisfactory data, which is symbolised by all the indicator blocks turning green^8^.

Strain elastography is a diagnostic method possessing some other technical limitations that may alter the results of the measurements. These limitations are particularly noticeable in methods employing an internal impulse to stimulate the tissue under study. In this case, the impulse power is difficult to control and standardize, and vectors of power from various sources (e.g., vibrations transmitted from the vessels, respiratory movements, and cardiac activity) may interfere with each other. In such cases, although physiological motion is required to perform the analysis, such motion also exhibits unpredictable variability, which may alter the outcome of the examination^1^. The impulse power may be influenced by the height of the pulse wave, the passage of the ultrasound wave through tissue barriers of various densities and the attenuation of the signal in the adipose tissue or amniotic fluid. In the literature on this subject, the results of the analysis of the correlation between the parameters obtained in the E-Cervix and baseline clinical factors are available. No correlation with the patient’s heart rate, mean arterial blood pressure, uterine artery pulsation or resistance index was found^10^. The variable significantly correlated with certain E-Cervix parameters in the quoted work was the patient's BMI, which was positively correlated with EOS and ECI readings and negatively correlated with HR. The correlation of BMI with several parameters (ECI and IOS) was also observed in our work. The correlation was very low (Spearman’s rho < 0.2) in both investigations^10^. In our study, we also demonstrated that several of the parameters may correlate with variables such as age (HR, EOS), number of previous CSs (HR) and patient weight (IOS/EOS). The observed relationship of HR with E-Cervix parameters may be the result of the degeneration of cervical connective tissue fibres under the influence of ageing and healing. The positive correlation of IOS/EOS parameters with weight may be a result of increased intra-abdominal pressure transferred to the internal cervical os in obese patients. In all cases, the correlation, although statistically significant, was very low (≤ 0.2), casting doubt on its clinical relevance; however, further research is required to assess the clinical implications. Similar relationships are observed in studies assessing the uterine cervix with SWE, and its stiffness measured with this method increases with age. In the cited study, no relationship was found between cervical stiffness and patient BMI^16^. Nevertheless, the analysis of correlation in our sample group may be subject to limitations due to the selection of the sample, which included patients hospitalized at the pregnancy pathology unit rather than a population of patients in physiological pregnancy.

The reproducibility assessment of a diagnostic method is always the foundation for research with higher clinical relevance. The quantitative result obtained on the basis of the elastogram allows a less subjective assessment of the uterine cervix compared to the semiquantitative scales typically used in strain elastography and creates an opportunity to compare results across research centres. The uterine cervix undergoes a natural process of softening as pregnancy progresses. There are initial reports in the literature regarding the sensitivity of the obtained E-Cervix parameters to physiological changes in cervical consistency for repeated measurements performed on the same pregnant patient as the pregnancy progresses^9^ Further natural development of this diagnostic method would involve its application in the prediction of preterm labour and the effectiveness of labour induction. Research demonstrates the usefulness of semiquantitative scales for these indications^7,17^, and there are initial reports of the use of E-Cervix for this purpose. In one of the reports published in 2019, the inclusion of the ECI parameter in the logistic regression model containing three other parameters (CL, prepregnancy BMI and gestational age at examination) for patients in the second trimester of pregnancy with cervix length ≥ 1.5 cm significantly increased the overall diagnostic accuracy of the test compared to screening based solely on CL^4^. In the perinatal period, some of the E-Cervix parameters (HR, IOS) in combination with CL allowed for increasing the predictive capacity of the model evaluating the chance of vaginal delivery in the next 24 h during labour induction with the use of a dinporoston vaginal insert^6^. Perhaps further research in elastography will contribute to its wider use in obstetrics. We are confident that the possibility of quantitatively evaluating the elastogram by the method studied by us, as well as the high repeatability we have obtained, are the features that will allow the performance of broader research in various clinical situations.

## Conclusion

All quantitative parameters in cervical elastography using the E-Cervix module are characterized by high repeatability and reproducibility. The obtained quantitative parameters correlate with some clinical features of the patients. The clinical relevance of this correlation requires further research.

## Data Availability

The dataset used for this study was uploaded to a public repository and is available at this URL: https://doi.org/10.17605/OSF.IO/6D9E7.
